# An intrauterine balloon for puerperal uterine inversion is not only for hemostasis but also for prophylaxis against reinversion: A letter to the editor

**DOI:** 10.1016/j.ijscr.2022.107397

**Published:** 2022-07-09

**Authors:** Shigeki Matsubara

**Affiliations:** Department of Obstetrics and Gynecology, Jichi Medical University, Tochigi, Japan,; Department of Obstetrics and Gynecology, Koga Red Cross Hospital, Koga, Ibaraki, Japan

**Keywords:** Condom catheter, Inversion, Reinversion, Uterine balloon

## Abstract

• Uterine inversion can recur after uterine repositioning.
• An intrauterine balloon prevents uterine reinversion.
• An intrauterine balloon should be inserted immediately after successful repositioning.

Dear Editor,

Puerperal uterine inversion causes massive hemorrhage. Inversion, after successful repositioning, can recur (reinversion), which causes life-threatening hemorrhage.

I congratulate Kurniawati [1] for accumulating knowledge on the usefulness of an intrauterine hemostatic balloon for uterine inversion: a condom catheter (one form of intrauterine balloon) achieved hemostasis. However, Kurniawati employed it for obstetric hemorrhage in general without specific consideration of uterine inversion or reinversion. I wish to share with the readers our view that an intrauterine balloon is useful not only for hemostasis but also for prophylaxis against reinversion.

Reinversion can occur after successful repositioning. We previously encountered a patient in whom reinversion occurred during a successful under-laparotomy repositioning procedure [2]. Data from Japan showed that ultrasound examination revealed that reinversion (including partial reinversion) occurred in 5/9 patients after successful repositioning [3].

In Kurniawati's patient, after successful manual uterine repositioning, physicians had to continue to perform “internal manual compression” to “maintain the repositioning”. However, re-bleeding occurred: a balloon was employed at this stage. This re-bleeding was possibly due to reinversion (including partial reinversion).

I wish to emphasize that special attention should be paid to balloon usage at inversion. An intrauterine balloon should be placed/employed earlier, i.e., just after successful repositioning, not after encountering re-bleeding. This is because a balloon prevents the occurrence of reinversion by compressing the uterine wall from inside (Fig. 1a versus 1b left). Additionally, earlier balloon placement might have freed an experienced doctor's hand from “manual compression”, thereby increasing available man-power at a critical period. Some physicians employ a balloon even at the time of the repositioning procedure: balloon insertion may help reposition an inverted uterus [4].

**Fig. 1:**
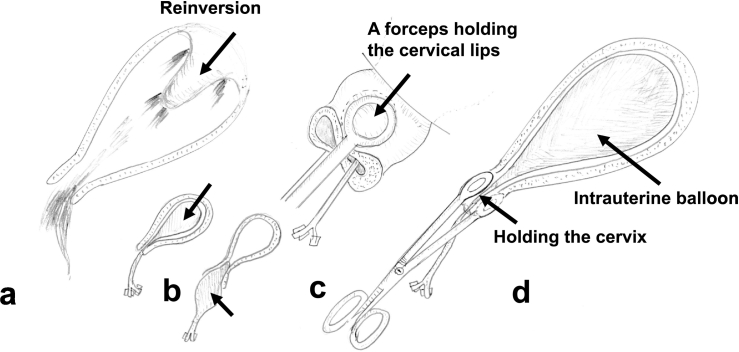
Schematic presentation of an intrauterine balloon plus the “holding the cervix” technique and its prophylactic effect against reinversion. a. The uterus, especially the uterine fundus, tends to reinvert (arrow) after successful repositioning. b. An intrauterine balloon is placed (arrow: left panel). This prevents the occurrence of reinversion and achieves hemostasis. The balloon often prolapses into the vagina (arrow: right panel). Inversion can recur. c. Both anterior and posterior cervical lips are held by sponge forceps, thereby closing the cervical canal. This prevents the balloon descending into the vagina (balloon prolapse). d. The intrauterine balloon continues to remain intrauterine, and thus it compresses the uterine luminal surface, achieving hemostasis, and, importantly, prevents the occurrence of reinversion.

I wish to touch on an additional procedure, which we usually use concomitantly with a balloon at the time of uterine inversion. An intrauterine balloon often prolapses into the vagina, causing balloon failure. To prevent this, we employ the “holding the cervix (Matsubara-Takahashi)” technique [5], [6]. Both anterior and posterior cervical lips are held/clamped by round forceps, thereby closing the cervical canal. This completely prevents balloon prolapse (Fig. 1). The balloon remains intrauterine, continuing to compress the uterine luminal surface, preventing the recurrence of uterine inversion. We have employed the holding the cervix technique for more than 200 patients, and have encountered no adverse events associated with this procedure [6], [7].

Uterine inversion can recur. An intrauterine balloon can prevent reinversion. I recommend balloon employment at an early stage of uterine inversion. Concomitant use of the holding the cervix technique may be considered.

## Funding

This letter to editor did not receive any specific grant from funding agencies in the public, commercial, or not-for-profit sectors.

## Ethical approval

None. This paper is in the format of letter to editor.

## Consent

None. This paper is in the format of letter to editor.

## Author contribution

Shigeki Matsubara: Reviewed the literature and wrote the manuscript.

## Registration of research studies

Not applicable.

## Guarantor

Shigeki Matsubara.

## Provenance and peer review

Not commissioned, externally peer-reviewed.

## Declaration of competing interest

None.
